# NEWS AND NOTICES

**Published:** 2017-02-10

## Next issue: online only

The next issue of the *Community Eye Health Journal* will be on **Neuro-ophthalmology.** It will include articles such as ‘Understanding vision and the brain’ and ‘Assessing the neuro-ophthalmology patient’. This issue will not be produced in paper format because of increasing costs in publication and distribution. It will be available online at **www.cehjournal.com** If you wish to receive an email with a link to download the PDF copy, please send your email address to **web@cehjournal.org.**

The next paper issue, planned for the end of March 2017, will be on **Continued Professional Development.** Thank you for your understanding.

## #StrongerTogether

**Figure:**
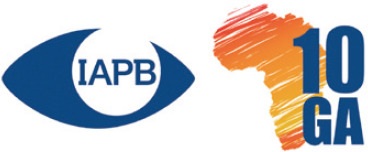


IAPB's General Assembly (10GA), the premier global event discussing public health issues related to blindness and visual impairment, brought together 1,150 eye care professionals from 100 countries in Durban in October 2016.

Over the course of three days, there were over 60 sessions with 200 speakers, and over 250 poster presentations. If you couldn't be there, you can catch up on what you've missed:

- View and download PowerPoint files of all the talks and presentations from **www.iapb.org/10ga-presentations**
- Access IAPB's Vision Atlas, which was launched at the 10GA. It allows access to the latest data and evidence related to avoidable blindness and sight loss: **http://atlas.iapb.org**
- Enjoy the photographs entered into the #StrongerTogether Photo Competition. The winners were announced at 10GA and all entries can be viewed at **http://photocomp.iapb.org**

## Courses

### German Jordanian University

Email: **vtc@gju.edu.jo**

### University of Cape Town Community Eye Health Institute

**www.health.uct.ac.za** or email **chervon.vanderross@uct.ac.za**

### Lions Medical Training Centre

Write to the Training Coordinator, Lions Medical Training Centre, Lions SightFirst Eye Hospital, PO Box 66576-00800, Nairobi, Kenya.

Tel: +254 20 418 32 39

### Kilimanjaro Centre for Community Ophthalmology International

Visit **www.kcco.net** or contact Genes Mng'anga at **genes@kcco.net**

## Subscriptions

**www.cehjournal.org/subscribe**

- For paper copies, email Anita Shah: **admin@cehjournal.org**
- To receive an alert when a new issue is published, email **web@cehjournal.org**

**Visit us online: www.cehjournal.org**

**www.facebook.com/CEHJournal/**

**https://twitter.com/CEHJournal**

