# Supplementary material for: NEWS AND NOTICES
Source: Community Eye Health. 2017 Feb 10;29(95):60. (PMC9112089)
Supplement: Related texts

# News: Useful resources for ocular surface disease

**Published:** 2016

## Cochrane reviews

### Blepharitis

Lindsley K, Matsumura S, Hatef E, Akpek EK. Interventions for chronic blepharitis. Cochrane Database Syst Rev. 2012, 5: CD005556. doi: 10.1002/14651858. CD005556.pub2

### Pterygium

Clearfield E, Muthappan V, Wang X, Kuo IC. Conjunctival autograft for pterygium. Cochrane Database Syst Rev. 2016, 2: CD011349. doi: 10.1002/14651858. CD011349.pub2.

### Dry Eye

Ervin AM, Wojciechowski R, Schein O. Punctal occlusion for dry eye syndrome. Cochrane Database Syst Rev. 2010, 9: CD006775. doi: 10.1002/14651858. CD006775.pub2.

### Herpes simplex

Wilhelmus KR. Antiviral treatment and other therapeutic interventions for herpes simplex virus epithelial keratitis. Cochrane Database Syst Rev. 2015, 1:CD002898. doi: 10.1002/14651858.CD002898.pub5.

### Squamous cell carcinoma

Gichuhi S, Irlam JH. Interventions for squamous cell carcinoma of the conjunctiva in HIV-infected individuals. Cochrane Database Syst Rev. 2013 28; 2:CD005643. doi: 10.1002/14651858. CD005643.pub3.

## Further reading

### Ocular surface

Gipson IK. The ocular surface: the challenge to enable and protect vision: the Friedenwald lecture. Invest Opht Vis Sci 2007;48(10):4390; 4391-4398.

Available online: **https://www.ncbi.nlm.nih.gov/pmc/articles/PMC2886589/**

### Dry eyes

Methodologies to diagnose and monitor dry eye disease: report of the Diagnostic Methodology Subcommittee of the International Dry Eye Workshop (2007). Ocul Surf. 2007;5(2):108-52. Available online: **www.tearfilm.org/dewsreport/**

Baudouin C, Messmer EM, et al. Revisiting the vicious circle of dry eye disease: a focus on the pathophysiology of meibomian gland dysfunction. BJO Online First, published on January 18, 2016. Available online: **http://tinyurl.com/dry-eye-circle**
